# Influenza epidemic surveillance and prediction based on electronic health record data from an out-of-hours general practitioner cooperative: model development and validation on 2003–2015 data

**DOI:** 10.1186/s12879-016-2175-x

**Published:** 2017-01-18

**Authors:** Barbara Michiels, Van Kinh Nguyen, Samuel Coenen, Philippe Ryckebosch, Nathalie Bossuyt, Niel Hens

**Affiliations:** 1 Department of Primary and Interdisciplinary Care Antwerp (ELIZA) - Centre for General Practice, Faculty of Medicine and Health Sciences, University of Antwerp, Antwerp, Belgium; 2 Department of Epidemiology, Faculty of Public Health, Ho Chi Minh University of Medicine and Pharmacy, Ho Chi Minh, Vietnam; 3 Systems Medicine of Infectious Diseases (SMID), Department of Systems Immunology, Helmholtz Centre for Infection Research, Braunschweig, Germany; 4 Vaccine & Infectious Disease Institute (VAXINFECTIO) - Laboratory of Medical Microbiology, Faculty of Medicine and Health Sciences, University of Antwerp, Antwerp, Belgium; 5 Epidemiology and Social Medicine (ESOC), Faculty of Medicine and Health Sciences, University of Antwerp, Antwerp, Belgium; 6 Unit Epidemiology of infectious diseases – Operational Directorate Public Health and Surveillance, Belgian Scientific Institute for Public Health, Brussels, Belgium; 7 Interuniversity Institute of Biostatistics and statistical Bioinformatics (iBIOSTAT), Hasselt University, Hasselt, Belgium; 8 Vaccine & Infectious Disease Institute (VAXINFECTIO) - Centre for Health Economic Research and Modelling Infectious Diseases, Faculty of Medicine and Health Sciences, University of Antwerp, Antwerp, Belgium

**Keywords:** Influenza-like illness, Influenza, Surveillance, Epidemics, Out-of-hours, Prediction, Epidemiology, Secular

## Abstract

**Background:**

Annual influenza epidemics significantly burden health care. Anticipating them allows for timely preparation. The Scientific Institute of Public Health in Belgium (WIV-ISP) monitors the incidence of influenza and influenza-like illnesses (ILIs) and reports on a weekly basis. General practitioners working in out-of-hour cooperatives (OOH GPCs) register diagnoses of ILIs in an instantly accessible electronic health record (EHR) system.

This article has two objectives: to explore the possibility of modelling seasonal influenza epidemics using EHR ILI data from the OOH GPC Deurne-Borgerhout, Belgium, and to attempt to develop a model accurately predicting new epidemics to complement the national influenza surveillance by WIV-ISP.

**Method:**

Validity of the OOH GPC data was assessed by comparing OOH GPC ILI data with WIV-ISP ILI data for the period 2003–2012 and using Pearson’s correlation. The best fitting prediction model based on OOH GPC data was developed on 2003–2012 data and validated on 2012–2015 data. A comparison of this model with other well-established surveillance methods was performed. A 1-week and one-season ahead prediction was formulated.

**Results:**

In the OOH GPC, 72,792 contacts were recorded from 2003 to 2012 and 31,844 from 2012 to 2015. The mean ILI diagnosis/week was 4.77 (IQR 3.00) and 3.44 (IQR 3.00) for the two periods respectively. Correlation between OOHs and WIV-ISP ILI incidence is high ranging from 0.83 up to 0.97. Adding a secular trend (5 year cycle) and using a first-order autoregressive modelling for the epidemic component together with the use of Poisson likelihood produced the best prediction results. The selected model had the best 1-week ahead prediction performance compared to existing surveillance methods. The prediction of the starting week was less accurate (±3 weeks) than the predicted duration of the next season.

**Conclusion:**

OOH GPC data can be used to predict influenza epidemics both accurately and fast 1-week and one-season ahead. It can also be used to complement the national influenza surveillance to anticipate optimal preparation.

## Background

Annual influenza epidemics induce heavy burdens on public health, including socio-economical and organizational [1]. Dealing with each seasonal influenza epidemic means an annual organizational challenge for health care systems. Timely information on an upcoming epidemic is essential to both optimising the organisation of manpower and medication stockpiling.

Worldwide, surveillance systems play a central role in supporting data-driven policies in public health intervention. In Belgium, this activity is organized by the Scientific Institute of Public Health (WIV-ISP) who provides weekly reports on the incidence of clinical influenza-like illness (ILI) and virological data collected by sentinel general practitioners (SGPs). Routine national surveillance data frequently have a reporting delay compared to real time incidents. Their primary goal is to announce the start/end of an influenza epidemic based on the trespassing of a certain incidence threshold and to document the impact of an ongoing influenza epidemic. Predicting future infuenza incidence is generally not included.

Establishing early detection and prediction systems is a crucial step to setting up effective control measures to combat upcoming epidemics. These systems rely primarily upon reliable and timely sources of data. In recent years, data that are electronically and routinely collected have emerged as convenient sources of surveillance data [2].

Health care is very often provided during out-of-hours services (OOHs) as this period accounts for more than two thirds of total care-time. In the last decade, the organization of OOHs in primary care in Flanders, Belgium improved dramatically through the on-going establishment of general practice cooperatives (GPCs). In 2003 Antwerp was the first region in Flanders to establish a GPC (Deurne-Borgerhout), which guided the establishment of many other GPCs. From the start, this GPC invested in producing high-quality, encoded, electronic health record (EHR) data.

Other European countries have benefited from such data collection initiatives. Data collected through the general practice OOHs have shown the early warning capability compared to the national surveillance system in Ireland [3]. Also, in Ireland and in Denmark the OOHs influenza-related calls peaked at least 1 week ahead of the national ILI rates [3, 4]. These findings illustrate the potential benefit of a regular analysis of ILI diagnoses registered on the spot by the OOH GPCs. Up to now no such analysis is performed and validated for future use in Belgium. Therefore in this paper we aim to develop a tool that can describe seasonal influenza epidemics earlier and as accurate as the national surveillance system and predict upcoming epidemics in the short and the long term based on OOH GPC EHR data on ILI. If successful, This tool can be implemented alongside the national influenza surveillance of the WIV-ISP and in GPCs spread all over the country to allow timely preparation for an upcoming epidemic by the different healthcare providers.

## Methods

### Data collection

#### OOH GPC data

**Fig. 1:**
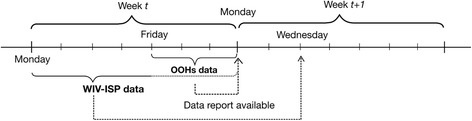
Data collecting and reporting of the OOH GPC and the national surveillance system (WIV-ISP)

The clinical data were collected in an EHR in the GPC Deurne-Borgerhout by the GPs on duty (about 100 each year) during the weekend from Friday evening 7 pm until Monday morning 7 am and on official holidays [5]. Deurne-Borgerhout is a part of the city of Antwerp, Belgium with more than 100,000 inhabitants. The catchment population covered by the GPC Deurne-Borgerhout was retrieved from the official website of the city of Antwerp, where the inhabitants of Deurne and Borgerhout were described and counted per year [6]. ILI diagnosis was based on the International Classification of Primary Care (ICPC)-2 code definition (R80) [7] and on the diagnostic study of Michiels et al. [8], i.e. a body temperature > 37.8 °C and cough must be present combined with other complaints such as headache, myalgia, fatigue, runny or stuffed nose and expectoration. The total number of consultations and the number of ILI diagnosed were retrieved per day. Data were generally available the first working day after the OOHs period, e.g. most commonly Monday after the weekend (Fig. 1).

#### WIV-ISP data

In Belgium, the influenza surveillance among the general population is performed by the National Influenza Centre, in collaboration with the Unit of Health Services Research and the Unit of Epidemiology of Infectious Diseases of the WIV-ISP in Brussels [9]. A network of 120 to150 SGPs, representing approximately 100,000–150,000 inhabitants.is involved in the clinical and virological influenza surveillance. The SGPs report on every patient with an ILI whom they have encountered during office hours and, occasionally during weekend OOHs, on a standardized paper form or by e-fax and on a weekly basis. The general criteria for ILI for the influenza surveillance are sudden onset of symptoms, high fever, respiratory (i.e. cough, sore throat) and systemic symptoms (headache, muscular pain) [10]. The aggregated results, integrated with the virological results, are available online on Wednesday of the week after the registration week (expressed as ISO week running fom Monday to the Sunday preceding the reporting date) (Fig. 1). Since no GP patient lists exist in Belgium, the average population coverage per GP (denominator) is estimated on the basis of the total Belgian population, divided by the total number of practising GPs in his region (based on figures from the National Institute for Health and Disability Insurance (NIHDI) [11]). The incidence is then estimated as the weekly number of ILI cases reported by the SGP divided by that denominator.

Data from both sources were collected retrospectively and anonymised before analysis. Ethics approval was granted by the Ethics Committee of the University of Antwerp for the retrospective use of OOH GCP data. Eligible patients were informed about the scientific goal of the clinical data collection. No written informed consent was collected.

The data collected were from 27^th^ June 2003 (week 26) to 23^rd^ March 2012 (week 12). They were used to assess the validity of OOH GPC data as a source of ILI surveillance and to develop a model for ILI epidemics (nine seasons). To validate the model (for three seasons), data were collected from 24^th^ March 2012 (week 13) to 16^th^ August 2015 (week 33).

### Validity of the OOH GPC data

To test the validity of OOHs data as a source for ILI surveillance, the estimated ILI incidence trends of the OOH GPC ILI data were compared with the trends of the WIV-ISP network by Pearson’s correlation coefficient within each epidemic season. ILI incidence per week is estimated by the number of reported cases with ILI symptoms in a certain week divided by the total number of consultations in that week. The difference with the denominator used by WIV-ISP in the ILI incidence calculation is no objection in the comparison of the trends as no exact match is required. However, this incidence estimate does not take into account the data of the other weeks, and provides no measures of variability around the estimated trends [2]. To alleviate these issues, a first-order random walk model (RW-1) was used to obtain smoother ILI incidence trends and the associated confidence bands.

### Model selection and validation

For the univariate time series of ILI counts {y_t_,t = 1,…,n},n = 634, the mean incidence was decomposed additively into an epidemic and an endemic component. The former is assumed to capture occasional outbreaks whereas the latter explains a baseline rate of cases with stable temporal pattern. The parametric model is given by

$$ \log \left({\mu}_t\right) = \left[{\beta}_0 + {\beta}_1t+{S}_t+{C}_s\right]+{\delta}_t+ \log \left({E}_t\right),\ t=1, \dots,\ n, $$

where β_0_ is the intercept; β_1_ t is the linear trend; S_t_ takes values s_t_ = -(s_(t-1)_ + ⋯ + s_(t-51)_),t = 52,53,…,n and represents the annual seasonal trend, C_s_ takes values c_s_ = -(c_(s-1)_ + ⋯ + c_(s-k)_), s = 2004,…,2015 and represents the secular trend every k years, k = 3,4,5; E_t_ is the total number of consultations at week t regardless of reasons. The terms in square brackets reflects the regular seasonal variation, δ_t_ represents the epidemic component. Poisson and Negative Binomial (NB) likelihood were considered for the ILI series. Different models of the epidemic component (δ_t_) were examined: (i) the independent and identically distributed (IID) model assumes independent effects across time; (ii) the RW-1 model implies dependence of the current value on the immediate past value; (iii) the first-order autoregressive (AR-1) model assumes a correlation between current and immediate past value (which reduces to RW-1 if this correlation is 1); and (iv) the second-order random walk (RW-2) model implies dependence on two previous time points. Sensitivity analyses of the prior choices for the hyperparameter of the epidemic component were performed. The priors considered included Gamma (1,0.01), Gamma (1,0.001), Gamma (1,0.00001), Gamma (1,0.00005), truncated Normal distribution HN(0,0.01), and HN(0,0.001). All the models were fitted using R-INLA package [12]. The Watanabe-Akaike information criteria (WAIC) [13], the logarithmic score [14] and the mean squared error (MSE) were used in combination to rank and select the best model for surveillance purposes. Here, the MSE reflecting the long-term prediction, was calculated as the average difference between the model prediction of the last three seasons and the corresponding observed data.

### Surveillance applications

To illustrate the surveillance application, the predictions of the best model are presented for the five full seasons from 2010 to 2015 together with the results obtained from the well-established methods using the surveillance package [15], including the methods that are currently employed by the Centers for Disease Control and Prevention (CDC) [16]; the Communicable Disease Surveillance Centre (CDSC) [17] and the Robert Koch Institute (RKI) [18]. To make the results comparable between methods, data on the first seven seasons were used as the default “past” data for each algorithm. The model developed for ILI counts was used to make two types of prediction: 1-week-ahead (OWA) and one-season-ahead (OSA) prediction. The OWA was calculated using the same approach as the Bayesian outbreak detection algorithm [19]. In short, the model predicts the ILI incidence of the immediate consecutive week, providing a threshold above which an alarm of aberrancy will be triggered whenever the observed ILI count exceeds this threshold. The threshold is the 97.5th percentile of the predictive posterior distribution. In the OSA prediction, the model predictions were made for the consecutive year, then the epidemic season indicators, including the start and the duration were calculated by the moving epidemic method [20]. In both OWA and OSA prediction, all the data up to but not including the week/season that is currently being predicted are used for model fitting.

## Results

### Data description and the validity of OOH GPC data

**Fig. 2:**
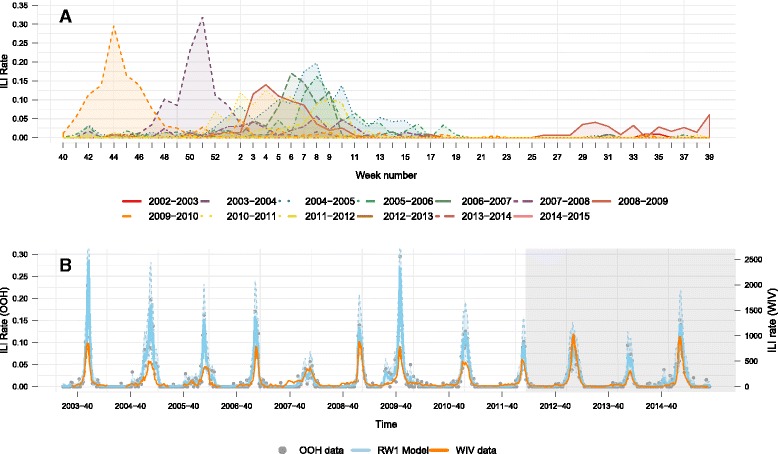
Data description and the validity of OOH GPC data. **a** Dynamics of the twelve influenza epidemic seasons from the OOH GPC data; **b** Estimated ILI incidence trends from the OOH GPC data (per total number of consultations) are shown along with the trends from the WIV-ISP data (per 100,000 persons). The *light blue band* presents the 95% credible interval of the estimated ILI incidence using the RW-1 model. The *darker area* indicates the data used for model validation

During the study period (2003–2012), there were 72,792 patient contacts recorded. Of the patients 43.9% were men and the mean age was 37.3 years. ILI was diagnosed in 2.2% of the cases. During the validation phase (2012–2015) 31,844 patient contacts were recorded, with a mean age of 36.9 years and of which 42.8% were men. The total number of inhabitants evolved from 111,011 in 2003, to 120,693 in 2012 and to 123,615 in 2015 [6]. The mean ILI diagnosis/week were 4.77 (IQR 3.00) and 3.44 (IQR 3.00) for the initial period until 2012 and the second period from 2012 to 2015, respectively. The ILI series exhibit a broadly regular pattern over years (Fig. 2a). Most often the epidemic season started on week 46, except for the pandemic in 2009–2010, and the epidemic began to die out after a 5 weeks increase. Then the epidemic reached the lowest activity period from week 20 onward. The first activity of a new season can be observed on week 30 with an exception for the pandemic in 2009. The epidemic seasons seem to follow a pattern that quickly increases at the beginning and slowly decreases with a somewhat longer tail to the right of the epidemic curve. Figure 2b presents the estimated OOH GPC ILI consultation trends together with the trends from the WIV-ISP. The two sources of data show a comparable course over years and a high correlation within each season, i.e. Pearson correlations for each epidemic season ranged from 0.83 to 0.97) (Table 1).

**Table 1:** Pearson correlations between ILI incidence from the OOH GPC and the WIV-ISP data

Season	OOH GPC vs WIV-ISP	RW-1 vs WIV-ISP
2003–2004	0.91 (34)	0.94 (34)
2004–2005	0.88 (38)	0.93 (38)
2005–2006	0.76 (37)	0.83 (37)
2006–2007	0.89 (52)	0.92 (52)
2007–2008	0.78 (52)	0.89 (52)
2008–2009	0.92 (52)	0.96 (52)
2009–2010	0.95 (52)	0.97 (52)
2010–2011	0.92 (52)	0.96 (52)
2011–2012	0.87 (52)	0.91 (52)
2012–2013	0.89 (52)	0.93 (52)
2013–2014	0.85 (52)	0.93 (52)
2014–2015	0.90 (46)	0.94 (46)

*OOH GPC* out-of-hours general practitioner cooperative, *RW-1* first order random walk model, *WIV-ISP* Scientific Institute of Public Health, Numbers in brackets are number of weeks recorded in both data

### The prediction model

Table 2 presents the best models from testing different model assumptions. The results show that the Poisson likelihood was preferred over the NB for the ILI series (Extended Table: see http://goo.gl/n5kHbU). Given the same model structure, the WAICs were consistently higher using the NB likelihood than using the Poisson likelihood. Epidemic component modelled with the first-order autoregressive (AR-1) was mostly better in different model structures. The three models M1, M3, M8 provided equivalent long-term prediction quality while their WAIC and logarithmic score are among the smallest. M8, the model with the simplest structure, was used for the surveillance application.

**Table 2:** Best models selected from fitting to the first nine seasons and the corresponding prediction error obtained from predictions for the last three seasons of the OOH GPC data

Epidemic	Endemic	k	logS^a^	WAIC^a^	MSE^b^	
AR(1)	*β*_0_ + *β*_1_ *t* + *S*_*t*_ + *C*_*s*_	5	64.8761	1101.893	13.8822	M1
AR(1)	*β*_0_ + *β*_1_ *t* + *S*_*t*_		64.9161	1103.298	17.8292	M2
AR(1)	*β*_0_ + *S*_*t*_ + *C*_*s*_	5	64.9217	1101.101	14.5707	M3
AR(1)	*β*_0_ + *S*_*t*_ + *C*_*s*_	4	64.8822	1104.608	17.1564	M4
AR(1)	*β*_0_ + *S*_*t*_		65.1286	1101.176	16.6048	M5
RW-1	*β*_1_ *t* + *S*_*t*_ + *C*_*s*_	5	64.5683	1103.741	21.1512	M6
AR(1)	*β*_1_ *t* + *S*_*t*_		64.6006	1103.310	20458.71	M7
AR(1)	*S*_*t*_ + *C*_*s*_	5	64.5406	1102.243	14.2192	M8
IID	*S*_*t*_		403.3084	984.414	17.1342	M9
AR(1)	*S*_*t*_		64.3999	1102.030	908.6617	M10
AR(1)	*β*_0_ + *β*_1_ *t* + sin(2*πt*/52) + cos(2*πt*/52)		1.4243	1108.511	22.7856	M11

All the presented models used Poisson likelihood for the ILI counts. An extended table can be found [http://goo.gl/n5kHbU]; ^a^Used the first nine seasons data, ^b^for the last three seasons data; *logS* logarithmic score, *WAIC* Watanabe-Akaike information criteria, *MSE* mean square error

### One week ahead and one season ahead prediction

**Fig. 3:**
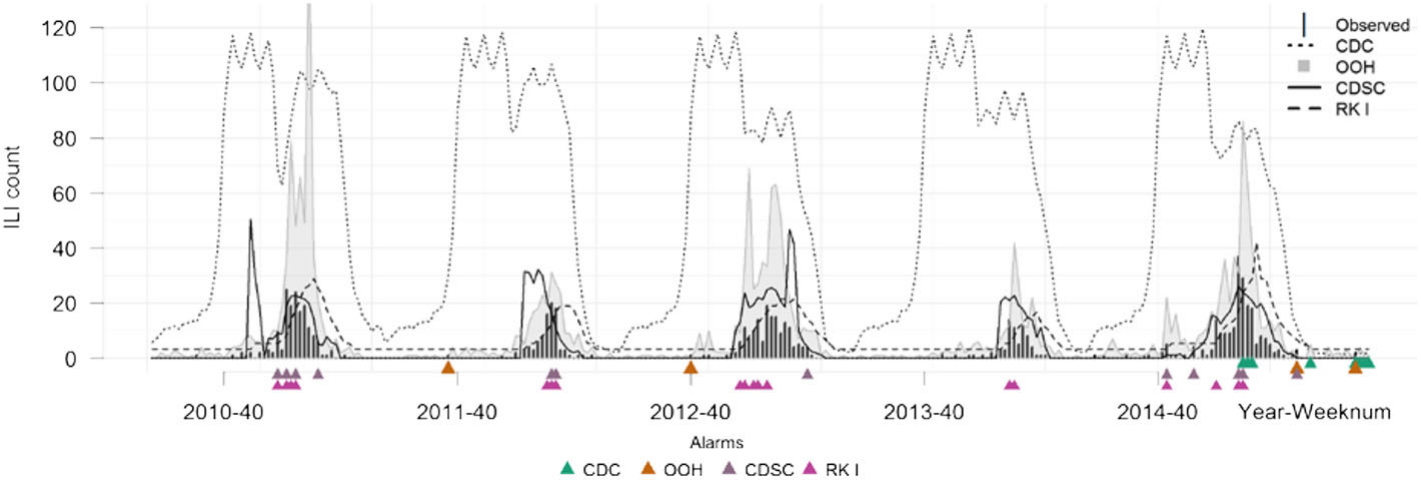
OOH GPC model and other algorithms: upperbound prediction’s and the corresponding alarms for five seasons (2010–2015). CDC: Centers for Disease Control and Prevention [16]; OOH: out-of-hours general practitioner collaborative Deurne-Borgerhout; CDSC: the Communicable Disease Surveillance Centre [17]; RKI the Robert Koch Institute [18]

Figure 3 illustrates the surveillance application, using the OOH GPC model (M8) and other existing algorithms to obtain the prediction’s upper bound and the corresponding alarms, showing that the real incidence is exceeding the predicted incidence, for the five full seasons from 2010 to 2015. The RKI’s upper bound loosely followed the real ILI dynamic and even less so the CDC’s. The CDSC’s upper bound exhibits departure from the real ILI pattern in the first two seasons but catches up in the latter three. The CDC’s upper bound is the highest and the RKI is the lowest. As a result, the RKI gave the highest number of alarms over seasons whilst there are fewer alarms from CDC. The OOH GPC model yielded the smallest number of alarms and they appeared either in the beginning or at the end of the season. All of the alarms obtained from CDC and RKI were triggered during the high intensity period of the epidemic.

The OOH GPC model (M8) was further used for OSA prediction of the ILI epidemic. The median predicted ILI rate for each season was obtained to calculate the epidemic properties as presented in Table 3. The peak week was predicted more accurately over time, but mostly more than 1 week late. The starting week, on the other hand, was predicted mostly 3 weeks earlier. The best prediction was observed in the prediction of the epidemics duration (see Table 3).

**Table 3:** Observed versus one-season-ahead predicted epidemics using OOH GPC ILI data

	Peak week^a^		Start^a^		End^a^		Duration^b^	
Season	Obs.	Pred.	Obs.	Pred.	Obs.	Pred.	Obs.	Pred.
2010–2011	2	8 (8–8)	47	41 (41–44)	12	18 (15–18)	18	30 (24–30)
2011–2012	9	7 (2–8)	1	50 (40–48)	18	15 (17–18)	18	18 (23–30)
2012–2013	5	8 (8–8)	45	42 (41–48)	15	13 (18–18)	23	24 (23–30)
2013–2014	7	8 (8–8)	50	44 (42–47)	15	15 (13–18)	18	24 (24–24)
2014–2015	6	7 (42–8)	47	44 (40–50)	16	13 (15–18)	22	22 (21–28)

^a^Week number; ^b^Number of weeks; *Obs.* observed data, *Pred.* prediction. Calculations are done as in [20]. The numbers in brackets are the values calculated by applying the calculation on 0.025 and, 0.975 percentiles of ILI prediction curves, respectively. Due to the small count, these curves are prone to distort the calculation

## Discussion

Based on ILI counts of nine influenza seasons (2003–2012) a prediction model was created taking into account an annual seasonal trend and most importantly a secular trend every 5 years. These proved to have excellent prediction capacities for both 1 week and one season ahead. Early detection of epidemics is a key element to prevent loss of (quality of) life and its economic and material impact.. In this study, the OOHs data from the GPC Deurne-Borgerhout reveals its attractive features that can facilitate an early detection of seasonal influenza epidemics. Their data are collected weekly, electronically recorded and readily available two days in advance of the WIV-ISP data. The time delay of WIV-ISP data reporting is mainly a consequence of the time needed for the virological confirmation required for WIV-ISP data. Importantly, the OOH GPC data showed remarkably high correlation with the nation-wide data. These results illustrate that data are not only credible but also advantageous to use for surveillance and prediction purposes, especially for an automatic detection system. GPC Deurne-Borgerhout is a small geographical area, yet its representativeness for the nation-wide data is striking. In the future, the extent of representation will be further improved when data are collected from more GPCs. It is worth mentioning that regardless of the lack of virological confirmation in the OOH GPC data, the high correlation underlines the accurateness of the used clinical diagnosis of influenza by GPs [8].

Many algorithms used for diseases surveillance are well-established; however, each method by some means is context- and disease-specific. This is because of the differences in surveillance purposes, the disease’s epidemiologic features, or the approach in calculating the alarm threshold. For instances, CDC and CDSC algorithm use a generic approach to monitor several pathogens at once [17], whereas the RKI algorithm uses different reference time points to calculate the threshold. ILI data however exhibit a broadly regular seasonal variation with the starting time of the epidemic season fluctuating every year, implying that a method relying solely on the fixed reference time points could be inadequate. Furthermore, secular trend is a would-be term in the model considering the recycling of influenza and the secular variations in population aging over the time course of the study [21]. To this end, we first used the Forecast library in R [22] to select the most appropriate forecasting method using the corrected Akaike information criteria (AICs). The resulting best-fit AR model yielded bad prediction quality in long-term prediction MSE because of which we moved to a Bayesian approach. In the Bayesian mode, we incorporated a secular trend along with seasonal variations to model the baseline ILI rate. The results showed that the models accounting for the secular trend were among the best models and provided better long-term predictions, suggesting influenza epidemics possess secular features. The epidemic component was also examined and appeared to be better modelled with AR-1, which agrees with the literature [19, 23, 24].

The model for the surveillance application (M8) was selected because of its similarity in structure with the better ones and its simplicity. It properly predicted the upcoming influenza epidemics both in the long- and short-term by providing early and closely warning alarms for the start of the epidemic seasons (Table 3, Fig. 3). This is further shown in the lower number of alarms in the epidemics periods (Fig. 3). Coherently, the more accurate the prediction model, the less alarms are generated. When alarms are generated, it means they are more likely to be an irregular but real incidence instead of data error. Therefore, an accurate prediction model will not only reduce the number of false alarms but also avoids raising alarms in an obvious high incidence period, preventing unnecessary additional resource mobilisation in practice. With the forthcoming data from others GPCs, further calibration of the current model for ILI will be orchestrated. In addition, the long-term prediction indicators (Table 3) would be better calculated using the moving epidemic method given a larger count of ILI incidences.

The OOH GPC data with its advantage in timeliness of reporting and the ease of access has the potential to be used in influenza outbreak surveillance systems besides the existing national influenza surveillance systems. In the future these OOH GPC data from several services in Flanders will be secured on a weekly basis in a large database called iCAREdata (Improving Care and Research Electronic Data Trust Antwerp), promising an even better source of surveillance data [25]. More than simple surveillance, which describes only the past, the OOH GPC data have the potential of accurate prediction in the short and the long term. Using a fast computing method, the surveillance model can be easily installed and fully implemented on the iCAREdata database. This would allow a prospective prediction of epidemics by using an automated query based on the described model. Validation of the prediction model using data from several OOH services will be performed when iCAREdata is fully operational. As such geographical differences could be further detected which is not possible on a national surveillance level.

## Conclusion

ILI counts instantly extracted from OOH GPC EHRs together with an accurately performing prediction tool based on past ILI trends have the potential of early and accurate influenza forecasting. Such reliable influenza forecasting allows the timely preparation of the health care system, which benefits patients, healthcare workers and society.

## Abbreviations

AR-1: First-order autoregressive
CDC: Centers for Disease Control and Prevention
CDSC: Communicable Disease Surveillance Centre
EHR: Electronic health record
GPC: General practitioners cooperative
GPs: General practitioners
iCAREdata: Improving Care and Research Electronic Data Trust Antwerp
ILI: Influenza-like illness
IQR: Interquartile range
MSE: Mean squared error
NB: Negative Binomial
OOH: Out-of-hours
OSA: One-season-ahead prediction
OWA: One-week-ahead prediction
RKI: Robert Koch Institute
RW-1: A first-order random walk model
SGPs: Sentinel general practitioners
WAIC: Watanabe-Akaike information criteria
WIV-ISP: Scientific Institute of Public Health in Belgium
